# A Practical Neighbor Discovery Framework for Wireless Sensor Networks

**DOI:** 10.3390/s19081887

**Published:** 2019-04-20

**Authors:** Zhaoquan Gu, Yuexuan Wang, Wei Shi, Zhihong Tian, Kui Ren, Francis C.M. Lau

**Affiliations:** 1 Cyberspace Institute of Advanced Technology, Guangzhou University, Guangzhou 510006, China; 2 College of Computer Science and Technology, Zhejiang University, Hangzhou 310027, China; 3 Department of Computer Science, The University of Hong Kong, Hong Kong, China; fcmlau@csis.hku.hk; 4 School of Information Technology, Carleton University, Ottawa, ON K1S5B6, Canada; wei.shi@carleton.ca; 5 Institute of Cyberspace Research, Zhejiang University, Hangzhou 310027, China; kuiren@zju.edu.cn

**Keywords:** neighbor discovery, wireless sensor networks, communication collision, latency, energy consumption

## Abstract

Neighbor discovery is a crucial operation frequently executed throughout the life cycle of a Wireless Sensor Network (WSN). Various protocols have been proposed to minimize the discovery latency or to prolong the lifetime of sensors. However, none of them have addressed that all the critical concerns stemming from real WSNs, including communication collisions, latency constraints and energy consumption limitations. In this paper, we propose Spear, the first practical neighbor discovery framework to meet all these requirements. Spear offers two new methods to reduce communication collisions, thus boosting the discovery rate of existing neighbor discovery protocols. Spear also takes into consideration latency constraints and facilitates timely adjustments in order to reduce the discovery latency. Spear offers two practical energy management methods that evidently prolong the lifetime of sensor nodes. Most importantly, Spear automatically improves the discovery results of existing discovery protocols, on which no modification is required. Beyond reporting details of different Spear modules, we also present experiment evaluations on several notable neighbor discovery protocols. Results show that Spear greatly improves the discovery rate from 33.0% to 99.2%, and prolongs the sensor nodes lifetime up to 6.47 times.

## 1. Introduction

With the ascent of Internet-of-Things (IoT) [1,2,3,4,5,6,7], wireless sensor networks (WSNs) are increasingly being adopted for tracking and monitoring applications in various areas such as health-caring, smart buildings, agricultural management and assisted living [8,9,10]. For example, WSN has been deployed for agriculture information monitoring [11], and sensors can be attached to inventory items in a large warehouse for object identification [12].

As a crucial process in constructing a wireless network, *neighbor discovery*, where sensor nodes try to find the existence of neighboring nodes within their communication range, has drawn much attention in the last decade or so [12,13,14,15,16,17,18,19,20,21,22,23,24,25,26,27]. Sensor nodes are powered by batteries. To minimize the overall energy consumption, most existing work focuses on designing discovery schedules that maintain a low *duty cycle*. However, despite decades of efforts, designing practical neighbor discovery protocols for real-life WSNs remains a big challenge, especially when considering all three critical factors as pointed out by previous works:

1. Collisions happen when multiple nodes transmit on the same communication channel simultaneously.
2. Bounded latency is required for time-sensitive applications. For example, object detection applications require discovering the neighbors for transmitting emergent information with very low latency.
3. Prolonged lifetime of sensor nodes. In order to reduce the energy consumption and prolong the lifetime of sensor nodes, affective energy management methods that can dynamically adjust nodes’ duty cycles during the discovery process are crucial.

Neglecting all these practical concerns, sensor nodes will likely fail to discover all neighbors before running out of energy.

**Figure 1:**
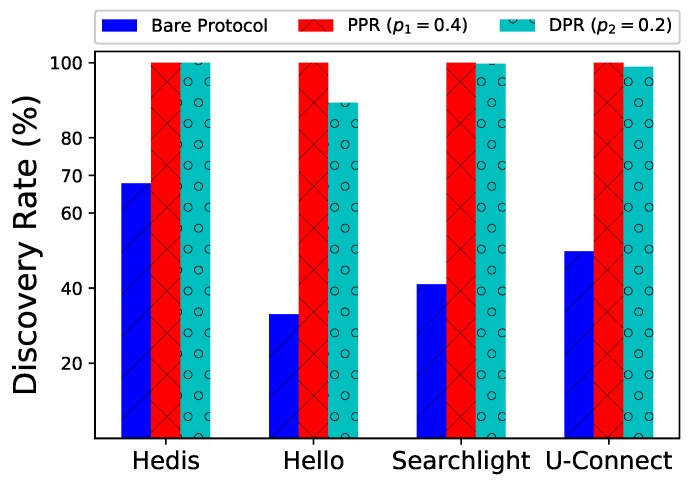
Spear greatly improves discovery rates for four notable protocols. Discovery rate for each protocol is defined as the number of discovered neighbors divided by all actual neighbors. Bare protocols mean they do not run in Spear.

Unfortunately, no existing work has considered all of the above-mentioned concerns in one setting. Typically, only one factor is considered at a time. Among all solutions, two major types of protocols exist: probability-based and deterministic ones. Probability-based protocols turn on the radio with different probabilities to reduce communication collisions [20,25,27,28,29], but the discovery latency often varies significantly. Compared to probability-based protocols, deterministic protocols are much more popular in practice. This approach is also the main focus of this paper because with a deterministic discovery schedule the discovery latency is mostly stable [12,13,15,16,18,21,22,26,30,31]. However, existing deterministic protocols mainly target at only two neighbors, where collision often does not occur. As shown in Figure 1, when these (bare) deterministic protocols (Hedis [15], Hello [22], Searchlight [13], and U-Connect [18]) are adopted in a network of 1000 nodes, the discovery rates are only 33.0–65.9% because of collisions.

In this paper, we propose **Spear** (Spear is a general, powerful ancient weapon), a practical neighbor discovery framework that deals with all the critical factors mentioned above. The advantages of Spear are:

(a) Spear creates two new methods, Pure Probability Reducing (PPR) and Decreased Probability Reducing (DPR), to reduce communication collisions among multiple nodes. Running in Spear, a deterministic protocol targeting at two neighbors can be automatically extended to support multiple nodes, while still keeping a stable discovery latency;
(b) Spear introduces two methods to manage energy and one unified method to handle latency constraints; it prolongs the node’s lifetime and enhances the availability for various applications;
(c) Spear enables the quantitative analysis of various neighbor discovery protocols, and generates the optimal neighbor discovery schedule automatically.

We implemented Spear and evaluated several notable neighbor discovery protocols on 1000 nodes. As shown in Figure 1, when running PPR or DPR in Spear, the discovery rates for these protocols greatly increase from 33.0% to 99.2%. By incorporating the energy management methods, nodes’ lifetime can be extended up to 6.47 times than that of running bare protocols.

The main contributions of Spear are automatic reduction of communication collisions, improvement of discovery rate, and prolonging lifetime for neighbor discovery, which together make the construction of WSNs much more effective and easier. Furthermore, Spear can be broadly applied to tackle various problems in WSNs.

The rest of the paper is organized as follows. We introduce relevant neighbor discovery protocols in Section 2, and the preliminaries in Section 3. We describe Spear in detail in Section 4. Methods that manage node energy and handle the latency requirements are presented in Section 5, and the methods to reduce communication collisions for an arbitrary neighbor discovery protocol are introduced in Section 6. We implemented Spear and evaluated several notable neighbor discovery protocols; the results are presented and discussed in Section 7. Finally, we conclude the paper in Section 8.

## 2. Related Works

The neighbor discovery problem in WSNs has been widely studied and the goal is to reduce the duty cycle or to reduce the latency of discovering the neighboring nodes. Generally speaking, there are two categories of neighbor discovery algorithms.

One category is *probability algorithms*, which utilize randomness to discover the neighbors in a short expected time. *Birthday protocol* [20] is one of the earliest algorithms that works on the *birthday paradox*, i.e., the probability that two people have the same birthday exceeds $\frac{1}{2}$ among 23 people. In the birthday protocol, each node transmits with probability p∈[0,1] and listens on the channel with probability 1−p in each time slot independently; this protocol ensures that the nodes can discover the neighbors with high probability, but it cannot deduce a bounded discovery time. Aloha-like protocol [28] assumes each node is awake in each slot with probability $p_{w}$ and an awake node transmits with probability $p_{t}$ and listens with probability $p_{l}$; one can derive the expected time to discover all neighbors with this protocol, but cannot guarantee successful discovery for the worst case situation. Following that, more smarter probabilistic algorithms were proposed [25,27,29], but they cannot guarantee an upper bound on the discovery latency among the nodes.

The other category is *deterministic algorithms*, which adopt some mathematic tools to ensure discovery between every two neighbors. The first tool is called *quorum system*: for any two intersected quorums, two neighboring nodes could choose any quorum in the system to design the discovery schedule and the discovery latency can be bounded in a short time. Many algorithms are related to the quorum system [15,17,18,19,30], but only a few of them support asymmetric duty cycles of the nodes, such as Hedis [15]. Another important tool is *co-primality* where two co-prime numbers are chosen by the neighbors to design the discovery schedule, and they can discover each other within a bounded latency by the Chinese Remainder Theorem [32]. Some representative algorithms are Disco [16], U-Connect [18], and Todis [15].

In short, probabilistic algorithms cannot guarantee two neighboring nodes discover each other in a finite time, but they assure that the discovery can be successful with a high probability in an expected discovery time. In contrast, deterministic algorithms can ensure the discovery process between the nodes, and they can limit the maximum discovery latency to within a bounded time.

Many neighbor discovery protocols assume that time is divided into slots of equal length and the nodes have aligned slots. Some works also study a general scenario that the slots are aligned between the users. They use *probe*, *beacon* or *anchor* to design the discovery protocols, and the representative algorithms are Searchlight [13], Hello [22] and Nihao [21]. There are also some other neighbor discovery protocols that are based on different techniques, such as combinatorial design [33], BlindDate [26], and Panda [12], the details of which we omit here. Among these algorithms, some of them only support *symmetric duty cycle* (the nodes select the same duty cycle), such as Quorum and Balanced Nihao [21], while others support asymmetric duty cycles (nodes select different duty cycles), such as Disco, U-Connect, Searchlight [13], Hello [22], Hedis and Todis [15].

To the best of our knowledge, most deterministic neighbor discovery algorithms are designed for two neighbors, with symmetric or asymmetric duty cycles. A few of them consider the energy management of each node and they ignore the communication collisions when they are extended for multiple nodes. Therefore, we propose a practical framework incorporating these issues and enable the existing deterministic protocols to be applicable for networks with multiple nodes.

## 3. Preliminaries

### 3.1. Sensor Node Model

Each sensor node $u_{i}$ has a distinguishable identifier $I_{i}$. Suppose node $u_{i}$ is powered by the battery it carries, we can denote the maximum power as $P_{max}$ and the remaining energy at time *t* as $P_{i}\left(t\right)$. The node dies at time *t* if $P_{i}\left(t\right)=0$ (rechargeable battery is a possibility but not considered here).

Driven by different applications, each node can carry out many operations, such as sensing nearby information, transmitting data, etc. Among these operations, communications through the wireless channel dominate the energy consumption [12]. Therefore, we assume only two states {ON,OFF} in this paper; OFF means the node turns its radio off to save energy, while ON means the node turns the radio on for communication. We focus on the communications during neighbor discovery process. Before each node tries to communicate with others, it has to identify the neighboring nodes first. Therefore, we suppose the nodes can communicate only when the discovery is successful.

**Figure 2:**
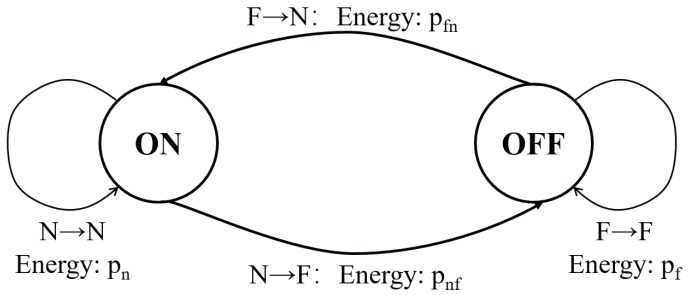
The finite state machine (FSM) of a sensor node’s states.

Suppose time is divided into slots of equal length $t_{0}$ which is sufficient for the nodes to establish a communication link on the channel. Denote the consumed energy in each time slot as $p_{n}$ if the node’s radio is turned on, and $p_{f}$ if the radio is turned off. Notice that a node may send a beacon message for discovering neighbors, listen on channels, exchange information, etc. Different operations may consume different energy when the radio is ON; we assume $p_{n}$ is the average energy in a time slot for simplicity. In practical systems, switching the radio states also consumes energy, such as $p_{nf}$ (switching the radio from on to off) and $p_{fn}$ (switching the radio from off to on). As shown in Figure 2, a node has two states {ON,OFF} and it switches states in each time slot. When a node switches from OFF to ON as F→N, the consumed energy is $p_{fn}$; when it switches from ON to OFF as N→F, the consumed energy is $p_{nf}$; when it keeps state ON as N→N, the (average) consumed energy in the slot is $p_{n}$; when it keeps state OFF as F→F, the consumed energy is $p_{f}$. In this paper, we adopt the common assumption $p_{f}=p_{nf}=p_{fn}=0$, and prolonging the lifetime of the node is equivalent to reducing its percentage of time slots when the radio is on. The notations are also given in Table 1.

### 3.2. Communication Model

Considering a WSN that consists of *N* nodes, $\left\{u_{1},u_{2},\ldots ,u_{N}\right\}$. Suppose only one wireless channel is available for communication. When the nodes turn on their radios simultaneously, they can transmit information through the wireless channel. Denote the communication range of each node as $d_{c}$, and two nodes are called *neighbors* if their distance is no larger than $d_{c}$ (denote the distance of nodes $u_{i},u_{j}$ as $d(u_{i},u_{j})$).

In real networks, whether one node can communicate with a neighboring node successfully is dependent on many factors, such as environment noises, the sending power energy, the path-loss exponent during transmission, beaconing, and handshaking. The signal-to-interference-plus-noise ratio (SINR) model is a realistic model that captures the collision among multiple transmissions [34]. It is also shown that the SINR model can be converted to the communication graph model that two nodes are neighbors if their distance is within the communication range. Therefore, we simplify the process and assume that two neighboring nodes can communicate if their distance is within $d_{c}$ and they both have turned on the radio.

In the practical networks, multiple nodes may turn on the radio simultaneously and they could cause communication collisions on the channel. For example, node $u_{1}$ has two neighbors $u_{2},u_{3}$ and they all turn on the radio simultaneously. Suppose both $u_{2},u_{3}$ send a message to $u_{1}$; then, $u_{1}$ cannot decode the composited message correctly. Therefore, we say *communication collision* happens and node $u_{1}$ cannot find its neighbors.

### 3.3. Neighbor Discovery

Neighbor discovery is the foundation of constructing WSNs. When the sensor nodes are deployed in the monitoring area, each node can only know its local information; the nodes have to find their neighboring nodes, and then the network can be established.

Suppose node $u_{i}$ starts at time $t_{i}^{s}$ and it tries to discover its neighbors by turning on the radio. In order to save energy, the node runs some pre-defined algorithms to generate a discovery schedule $S_{i}=\left\{s_{i}\left(t\right)|t\geq t_{i}^{s}\right\}$, where:

$$\begin{array}{c} s_{i}\left(t\right)=\left\{\begin{array}{cc} 0, & if\;u_{i}\;turns\;the\;radio\;OFF,\\ 1, & if\;u_{i}\;turns\;the\;radio\;ON. \end{array}\right. \end{array}$$

The **neighbor discovery** problem between two neighboring nodes is defined as:

**Problem** **1.** *For two neighboring nodes $u_{i}$ and $u_{j}$, design the discovery schedules $S_{i},S_{j}$ respectively such that there exists T satisfying:*

$$s_{i}\left(T\right)=s_{j}\left(T\right)=1.$$

Two nodes may start at different times, which is referred to as the *asynchronous* case in the literature, and the *discovery latency* is defined as:

**Definition** **1.** *The discovery latency between two neighboring nodes $u_{i}$ and $u_{j}$ is the time cost to turn on the radio simultaneously after they both have started:*

(1) $$L\left(i,j\right)=T-\max\left\{t_{i}^{s},t_{j}^{s}\right\}.$$

Considering the network with multiple nodes, the neighbor discovery problem for node $u_{i}$ is defined as:

**Problem** **2.** *For each node $u_{i}$, denote the set of neighboring nodes as $N_{i}=\left\{u_{j}|d\left(u_{i},u_{j}\right)\leq d_{c}\right\}$. Design the discovery schedule for each node, such that there exists $T_{i,j}$ satisfying:*

$$\begin{array}{c} \left\{\begin{array}{c} s_{i}\left(T_{i,j}\right)=s_{j}\left(T_{i,j}\right)=1,\\ s_{k}\left(T_{i,j}\right)=0,\forall u_{k}\in N_{i},u_{k}\neq u_{j}. \end{array}\right. \end{array}$$

Similarly, the *discovery latency* for node $u_{i}$ is defined as:

**Definition** **2.** *The discovery latency for node $u_{i}$ is the time cost to find all neighbors:*

(2) $$L\left(i,N_{i}\right)=\max_{u_{k}\in N_{i}}T_{i,k}-t_{i}^{s}.$$

Most neighbor discovery algorithms generate their discovery schedules with regard to the *duty cycle* which is defined as:

**Definition** **3.** *The duty cycle of node $u_{i}$ between time $T_{1},T_{2}$ ($T_{1}<T_{2}$) is the percentage of time slots when $u_{i}$ turns on the radio:*

$$\theta_{i}\left(T_{1},T_{2}\right)=\frac{\left|\{s_{i}\left(t\right)=1|T_{1}\leq t\leq T_{2}\}\right|}{T_{2}-T_{1}}.$$

The existing deterministic algorithms try to minimize the discovery latency between two neighbors for pre-defined duty cycles. Both symmetric and asymmetric duty cycles should be considered.

## 4. Spear: Neighbor Discovery Framework

### 4.1. Framework Overview

**Figure 3:**
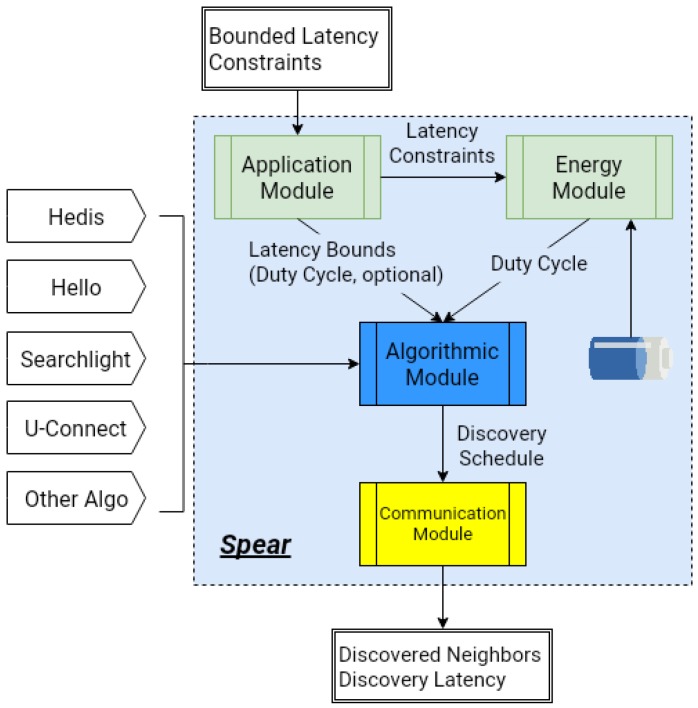
Overview of Spear.

As shown in Figure 3, Spear consists of four modules: **energy module** is in charge of the node’s energy management; **application module** collects various latency constraints from the applications; **algorithmic module** generates a neighbor discovery schedule by invoking the discovery protocols; and **communication module** is responsible for the communication with neighbors.

Spear accepts bounded latency constraints and remaining energy as the inputs, and the neighbor discovery algorithms (such as Hedis, Hello, Searchlight, and U-Connect in the figure) are plugged into the framework to generate the discovery schedule. Spear outputs the discovered neighbors and the corresponding discovery latency, which both are needed for constructing the network and achieving other functions.

### 4.2. Energy Module

The energy module receives the remaining energy and the latency constraints from the application module as inputs. The output is the duty cycle which the node is to adopt. Two main functions are incorporated into the energy module: *computing the node’s lifetime* and *adjusting the duty cycle*.

For any node $u_{i}$ which starts at time $t_{i}^{s}$, suppose the generated schedule is $S_{i}=\left\{s_{i}\left(t\right)|t\geq t_{i}^{s}\right\}$ and it runs out of energy at time $t_{i}^{e}$. We have the following equation:

(3) $$\int_{t_{i}^{s}}^{t_{i}^{e}}s_{i}\left(t\right)\cdot p_{n}=P_{max}.$$

Then, the lifetime of node $u_{i}$ (denoted as $Lf_{i}$) is $Lf_{i}=t_{i}^{e}-t_{i}^{s}$.

Notice that we assume a sensor node has only two states {ON,OFF} in Section 3.1. The generated schedule $S_{i}=\left\{s_{i}\left(t\right)\right\}$ contains a sensor state of each time slot *t*; specifically, $s_{i}\left(t\right)=0$ if the state of the node is OFF while $s_{i}\left(t\right)=1$ is the state of the node is ON. We define two identification functions as $f_{n2f}\left(t\right)=1$ if and only if $s_{i}\left(t-1\right)=1,s_{i}\left(t\right)=0$, $f_{f2n}\left(t\right)=1$ if and only if $s_{i}\left(t-1\right)=0,s_{i}\left(t\right)=1$. The first one implies that the node switches its state from ON to OFF, while the other one implies that the node switches its state from OFF to ON. Combining these, a complete energy formulation should be

$$\int_{t_{i}^{s}}^{t_{i}^{e}}s_{i}\left(t\right)\cdot p_{n}+\left(1-s_{i}\left(t\right)\right)\cdot p_{f}+f_{n2f}\left(t\right)\cdot p_{nf}+f_{f2n}\left(t\right)\cdot p_{fn}=P_{max}.$$

Since we assume the consumed energy of state OFF and switching states are zero, i.e., $p_{f}=p_{nf}=p_{fn}=0$, we derive the simplified formulation as Equation (3).

If node $u_{i}$ turns the radio on all the time, Equation (3) can be rewritten as:

$$\left(t_{i}^{e}-t_{i}^{s}\right)\cdot p_{n}=P_{max}$$

and the lifetime is $Lf_{i}=\frac{P_{max}}{p_{n}}$, which is the minimum value.

To extend the lifetime, node $u_{i}$ turns on the radio for a fraction of the time. If node $u_{i}$ selects the duty cycle as a fixed value $\hat{\theta }\in \left(0,1\right)$, we can rewrite Equation (3) as:

$$\left(t_{i}^{e}-t_{i}^{s}\right)\cdot \hat{\theta }\cdot p_{n}+\left(t_{i}^{e}-t_{i}^{s}\right)\cdot >(1-\hat{\theta }>)\cdot p_{f}\approx P_{max}.$$

We use ‘≈’ since the schedule may not be a complete cycle, but it makes very little difference, and we can just regard it as ‘=’. As we assume $p_{f}=0$, the lifetime of node $u_{i}$ is computed as:

(4) $$Lf_{i}=\frac{P_{max}}{p_{n}\cdot \hat{\theta }}.$$

Suppose that node $u_{i}$ adjusts the duty cycle timely. Denote the time that $u_{i}$ changes the duty cycle as: $T_{0}<T_{1}<\ldots <T_{m}$, where $T_{0}=t_{i}^{s}$ and $T_{m}<t_{i}^{e}$. For simplicity, denote $T_{m+1}=t_{i}^{e}$ and Equation (3) is rewritten as:

$$\begin{array}{c} \begin{array}{cc} & \sum_{k=0}^{m}\left(\int_{T_{k}}^{T_{k+1}}s_{i}\left(t\right)\cdot p_{n}\right)=P_{max}\\ \Rightarrow & \sum_{k=0}^{m}\left(T_{k+1}-T_{k}\right)\cdot \theta_{i}\left(T_{k},T_{k+1}\right)\cdot p_{n}=P_{max}. \end{array} \end{array}$$

Since $\theta_{i}\left(T_{k},T_{k+1}\right)$ and $T_{i},i\in \left[0,m\right]$ are known beforehand, $t_{i}^{e}=T_{m+1}$ is computed as:

(5) $$t_{i}^{e}=\frac{\frac{P_{max}}{p_{n}}-\sum_{k=0}^{m-1}\left(T_{k+1}-T_{k}\right)\cdot \theta_{i}\left(T_{k},T_{k+1}\right)}{\theta_{i}\left(T_{m},T_{m+1}\right)}+T_{m}.$$

Then, the lifetime can be computed. In Section 5, we analyze the impact of the node’s lifetime by different strategies that adjust the duty cycles.

### 4.3. Application Module

WSNs are used in many applications and there could be many different requirements for the nodes. For example, when a node detects an emergency, such as a very low temperature, a moving enemy, etc., it needs to inform the whole network quickly. We regard these requirements as latency constraints. That is, the node has to discover the neighbors within a bounded latency. Therefore, in order to send out the information quickly, the node has to increase the duty cycle and turn on the radio more frequently. The application module passes the latency constraints to the energy module and the algorithmic module. Two main functions are implemented in the module (for node $u_{i}$):

(1) To collect latency constraints at time *t*, such that the discovery latency should be bounded within ${\hat{L}}_{i}\left(t\right)$;
(2) to compute an appropriate duty cycle according to the latency constraint.

### 4.4. Algorithmic Module

Once the duty cycle is adjusted by the energy module or the application module, the node has to invoke the algorithmic module to compute the discovery schedule for the coming time slots. The interface involves duty cycle and latency constraints as inputs, and outputs the discovery schedule. Notice that Spear is designed for the practical networks and the nodes could adjust the duty cycle locally. Therefore, the implemented algorithms should be applicable for asymmetric duty cycles. We summarize the state-of-the-art algorithms by considering the relationship between duty cycles and discovery latency in Table 2.

### 4.5. Communication Module

The node carries out the operations according to the generated schedule by the algorithmic module. The target of the communication module is to discover the neighbors when collisions exist among multiple nodes. We summarize the two main functions that are implemented:

(1) Discover the neighbors and record the neighbors’ information, such as the identifier, the start time, and the duty cycle;
(2) compute the corresponding discovery latency of the neighbors.

When we deploy existing algorithms for multiple nodes, communication collisions often occur and many nodes cannot discover their neighbors. In Section 6, we devise two new methods to reduce the collisions, and the algorithms modified by the methods can achieve good performances.

### 4.6. Measurements

In this paper, we utilize three metrics to evaluate the algorithms. Considering each node $u_{i}$,

(1) **Lifetime**$Lf_{i}$ reveals how long the node can survive;
(2) **Discovery latency**$L(i,N_{i})$ is the number of time slots ($t_{0}$) to discover all neighbors;
(3) **Discovery rate** is the percentage of discovered neighbors in $N_{i}$ for a bounded latency.

Lifetime and discovery latency are commonly adopted in the existing works. Due to communication collisions, some nodes may not be able to find all neighbors, and so we introduce discovery rate for evaluation.

## 5. Methods of Adjusting Duty Cycle

Neighbor discovery is affected by nodes’ duty cycles. Existing works have designed efficient discovery schedules for fixed duty cycles, but few of them study how to adjust the duty cycle during a node’s lifetime. In this section, we present several methods to adjust the duty cycle according to the remaining energy and the latency constraints.

### 5.1. Energy Management Methods

As shown in Equation (4), node $u_{i}$’s lifetime is $Lf_{i}=\frac{P_{max}}{p_{n}\cdot \theta }$ if it sticks to duty cycle θ all the time. To extend the lifetime, it could reduce the duty cycle when the remaining energy is depleting. We propose two methods to adjust the duty cycle.

**Piece-wise Reducing (PWR) Method**: Generate *m* different energy levels as $P_{max}={\hat{P}}_{1}>{\hat{P}}_{2}>\ldots {\hat{P}}_{m}>0$ and *m* corresponding duty cycle levels as ${\hat{\theta }}_{1}>{\hat{\theta }}_{2}>\ldots >{\hat{\theta }}_{m}$ in advance. When the remaining energy drops down to ${\hat{P}}_{j}$, the node adjusts the duty cycle to ${\hat{\theta }}_{j}$. For simplicity, denote ${\hat{P}}_{m+1}=0$ and node $u_{i}$ selects duty cycle at time *t* as:

(6) $$\theta_{i}\left(t\right)={\hat{\theta }}_{j},\;if\;P_{i}\left(t\right)\in \left({\hat{P}}_{j+1},{\hat{P}}_{j}\right].$$

The lifetime of node $u_{i}$ is computed as:

(7) $$\begin{array}{c} \begin{array}{cc} Lf_{i} & =\sum_{j=1}^{m}\frac{{\hat{P}}_{j}-{\hat{P}}_{j+1}}{p_{n}\cdot {\hat{\theta }}_{j}}. \end{array} \end{array}$$

The PWR method can extend the node’s lifetime as compared to Equation (4), but it has to generate different levels of energy and duty cycles beforehand. We propose another method which is much easier to implement.

**Periodical Reducing (PDR) Method**: Node $u_{i}$ selects an initial duty cycle ${\hat{\theta }}_{0}$ when it starts (with energy $P_{max}$). The node adjusts the duty cycle every $\hat{T}$ time slots according to the remaining energy, where $\hat{T}$ is a fixed constant. Supposing that the remaining energy at time *t* is $P_{i}\left(t\right)$, the duty cycle is reduced as:

(8) $$\frac{P_{i}\left(t\right)}{\theta_{i}\left(t\right)}=\frac{P_{max}}{{\hat{\theta }}_{0}}.$$

When the remaining energy is very low ($P_{i}\left(t\right)\leq P_{min}$ where $P_{min}$ is a small constant), the node has to fix the duty cycle as ${\hat{\theta }}_{min}=\frac{P_{min}}{P_{max}}\cdot {\hat{\theta }}_{0}$. In order to compute the lifetime, it is necessary to compute the number of times that the node adjusts the duty cycle. Suppose after *m* periods of length $\hat{T}$, the remaining energy is no larger than $P_{min}$, and the following equations are derived:

(9) $$\begin{array}{c} \left\{\begin{array}{cc} {\hat{P}}_{0}=P_{max}, & {\hat{\theta }}_{0}={\hat{\theta }}_{0},\\ {\hat{P}}_{1}={\hat{P}}_{0}-\hat{T}\cdot {\hat{\theta }}_{0}\cdot p_{n}, & {\hat{\theta }}_{1}=\frac{{\hat{P}}_{1}}{{\hat{P}}_{0}}\cdot {\hat{\theta }}_{0},\\ \;\;\;\;\;\;\vdots & \;\;\;\;\;\;\vdots \\ {\hat{P}}_{i}={\hat{P}}_{i-1}-\hat{T}\cdot {\hat{\theta }}_{i-1}{\hat{p}}_{n}, & {\hat{\theta }}_{i}=\frac{{\hat{P}}_{i}}{{\hat{P}}_{0}}\cdot {\hat{\theta }}_{0},\\ \;\;\;\;\;\;\vdots & \;\;\;\;\;\;\vdots \\ {\hat{P}}_{m}={\hat{P}}_{m-1}-\hat{T}\cdot {\hat{\theta }}_{m-1}\cdot p_{n}, & {\hat{\theta }}_{m}=\frac{P_{min}}{{\hat{P}}_{0}}\cdot {\hat{\theta }}_{0}. \end{array}\right. \end{array}$$

Combine these to give:

(10) P^m=P^0[1−m1T^θ^0pnP^0+m2(T^θ^0pnP^0)2+⋯+(−1)mmm(T^θ^0pnP^0)m].=P^0(1−T^θ^0pnP^0)m.

When ${\hat{P}}_{m}\leq P_{min}$, the number of periods is $m\geq \frac{\log(P_{min}/P_{max})}{\log(1-\hat{T}{\hat{\theta }}_{0}p_{n}/P_{max})}$ and the lifetime of node $u_{i}$ is:

(11) $$Lf_{i}=m\cdot T+\frac{{\hat{P}}_{m}P_{max}}{P_{min}{\hat{\theta }}_{0}p_{n}}.$$

Both PWR and PDR methods could prolong the node’s lifetime and we evaluate them in Section 7.

### 5.2. Latency Constraints

When the applications have latency constraints, such as fast streaming or real time detection applications, the node has to increase the duty cycle in order to discover the neighbors in bounded time. However, discovery latency between two neighbors is determined by the chosen algorithm and the duty cycles of both nodes.

As listed in Table 2, different algorithms lead to different discovery latencies. Take Disco [16] as an example. Given latency constraint ${\hat{L}}_{i}\left(t\right)$ at time *t* for node $u_{i}$, it can check the recorded information of the discovered neighbors. Suppose one neighbor $u_{j}$’s duty cycle is ${\hat{\theta }}_{j}$, node $u_{i}$ has to increase its duty cycle as: ${\hat{\theta }}_{i}\geq \frac{4}{{\hat{\theta }}_{j}{\hat{L}}_{i}\left(t\right)}$. In order to discover all neighbors, node $u_{i}$ has to check the smallest duty cycle (denote it as ${\hat{\theta }}_{m}$) and it has to increase the duty cycle as ${\hat{\theta }}_{i}\geq \frac{4}{{\hat{\theta }}_{m}{\hat{L}}_{i}\left(t\right)}$. After satisfying the application requirements, node $u_{i}$ can then adjust the duty cycle by the remaining energy as described above. In order to reduce communication collisions, the duty cycle has to be larger.

Combining remaining energy and latency constraints, the duty cycle should be adjusted by both factors; that is, to design a function of adjusting the duty cycle as:

(12) $$\theta_{i}\left(t\right)=f\left(P_{i}\left(t\right),{\hat{L}}_{i}\left(t\right)\right).$$

When there is no latency constraint, ${\hat{L}}_{i}\left(t\right)=+\infty$, PWR and PDR are two representative examples. In Spear, researchers could implement the interface for evaluating more functions which can timely adjust the duty cycle.

## 6. Methods of Reducing Collisions

In real communication scenarios, two neighboring nodes can communicate successfully only when they are not interfered by other nodes. If existing deterministic algorithms are extended to handle multiple nodes directly, communication collisions happen and most nodes cannot find the neighbors. In this section, we analyze the discovery probability and propose two methods to reduce the collisions.

### 6.1. Discovery Probability under Collision

Considering two neighboring nodes $u_{i},u_{j}$, denote the sets of each node’s neighbors as $N_{i},N_{j}$ respectively ($u_{i}\in N_{j},u_{j}\in N_{i}$). Suppose that nodes $u_{i},u_{j}$ turn on their radio at time *t*, and denote the sets of neighbors that also turn on the radio as $\tilde{N_{i}}\subseteq N_{i},\tilde{N_{j}}\subseteq N_{j}$. Since $u_{j}\in \tilde{N_{i}},u_{i}\in \tilde{N_{j}}$, $u_{i}$ and $u_{j}$ can discover each other only when:

$$|\tilde{N_{i}}\left|=1,\right|\tilde{N_{j}}|=1.$$

Denote the average duty cycle for node $u_{k}$ as $\theta_{k}$. We consider the scenario where node $u_{k}$ turns on the radio with probability $\theta_{k}$ independently in each time slot (expected situation). Then, on the basis of the event that nodes $u_{i},u_{j}$ turn on the radio at time *t*, the probability for successful discovery is derived as:

$$\begin{array}{c} \begin{array}{cc} Pr(|\tilde{N_{i}}|=1,|\tilde{N_{j}}|=1) & \leq \min\{Pr(|\tilde{N_{i}}\left|=1),Pr(\right|\tilde{N_{j}}\left|=1)\right\}\\ & =\min\{\prod_{u_{k}\in N_{i},k\neq j}\left(1-\theta_{k}\right),\prod_{u_{k}\in N_{j},k\neq i}\left(1-\theta_{k}\right)\}. \end{array} \end{array}$$

If $\theta_{k}=1\%$ and $\max\{|N_{i}|,|N_{j}|\}\geq 69$ (or $\theta_{k}=10\%$ and $\max\{|N_{i}|,|N_{j}|\}\geq 7$), the probability of successful discovery is less than 1/2. Therefore, the existing algorithms cannot be applied to multiple nodes directly. We adopt the idea of the probabilistic protocols to reduce the communication collisions; two efficient methods are proposed.

### 6.2. Pure Probability Reducing (PPR) Method

The PPR Method works as follows. For any deterministic neighbor discovery algorithm *f*, denote the generated discovery schedule for node $u_{i}$ as $S_{i}=\left\{s_{i}\left(t\right)|t\geq t_{i}^{s}\right\}$. For any time *t* that $s_{i}\left(t\right)=1$, node $u_{i}$ turns on the radio with probability $p_{1}$ (a constant value in (0,1)); that is, to generate a modified sequence ${\tilde{S}}_{i}=\left\{{\tilde{s}}_{i}\left(t\right)|t\geq t_{i}^{s}\right\}$ as:

$$\begin{array}{c} \left\{\begin{array}{cc} If\;s_{i}\left(t\right)=0, & {\tilde{s}}_{i}\left(t\right)=0,\\ If\;s_{i}\left(t\right)=1, & {\tilde{s}}_{i}\left(t\right)=1\;with\;probability\;p_{1}. \end{array}\right. \end{array}$$

If two neighbors $u_{i},u_{j}$ turn on the radio at time *t*, the expected probabilities of $|{\tilde{N}}_{i}|=1$ and $|{\tilde{N}}_{j}|=1$ are:

$$\begin{array}{c} \begin{array}{cc} Pr(|\tilde{N_{i}}|=1) & =\prod_{u_{k}\in N_{i},k\neq j}\left(1-p_{1}\cdot \theta_{k}\right),\\ Pr(|\tilde{N_{j}}|=1) & =\prod_{u_{k}\in N_{j},k\neq i}\left(1-p_{1}\cdot \theta_{k}\right). \end{array} \end{array}$$

If $p_{1}=0.5$, $\theta_{k}=1\%$, the probability of successful discovery is less than 1/2 when $\max\{|N_{i}|,|N_{j}|\}\geq 139$. By choosing different values of $p_{1}$, the performance could be different. We evaluate the sensitivity of $p_{1}$ in Section 7.

### 6.3. Decreased Probability Reducing (DPR) Method

The PPR method can increase the discovery probability, but it is independent of the schedule itself. We present the DPR method, which tries to coordinate any discovery schedule with the method. For the generated discovery schedule $S_{i}$ by any algorithm *f*, node $u_{i}$ should turn on the radio at time $t_{1}$ when $s_{i}\left(t_{1}\right)=1$. Denote the next time slot that $u_{i}$ turns on the radio by schedule $S_{i}$ as $t_{2}$, i.e., $s_{i}\left(t_{2}\right)=1,t_{2}>t_{1}$. Modify the schedule of time slots $\left[t_{1},t_{2}\right)$ as:

- Change $s_{i}\left(t\right)=0$ for $t\in [t_{1},t_{2})$;
- increase $t^{*}$ from $t_{1}$ to $t_{2}-1$, if $s_{i}\left(t\right)=0$ for all $t\in [t_{1},t^{*})$, set $s_{i}\left(t^{*}\right)=1$ with probability $p_{2}\cdot \frac{t_{2}-t^{*}}{t_{2}-t_{1}+1}$ where $p_{2}$ is a constant value in (0,1).

Node $u_{i}$ turns on the radio in the initial slot with probability $p_{2}\cdot \left(1-\frac{1}{t_{2}-t_{1}+1}\right)$, and it could reduce the probability of collisions. If $u_{i}$ does not turn on the radio at time $t_{1}$, it decreases the probability and attempts to turn on the radio in the next slot, $t_{1}+1$. This process does not finish until $u_{i}$ turns on the radio in any slot within $\left[t_{1},t_{2}\right)$, or it keeps the radio off for all of them.

Overall, both PPR and DPR are designed to support deterministic neighbor discovery protocols. Unlike probabilistic-based protocols, the neighbor discovery schedules computed by both PPR and DPR are based on the schedules generated by deterministic protocols. Therefore, a deterministic protocol running with PPR or DPR can achieve a stable discovery latency (confirmed in Section 7.1).

## 7. Evaluations

We have implemented Spear in C++, which includes the interfaces between different modules, important functions for computing in each module, and measurements to evaluate the performances. We implemented four state-of-the-art algorithms including Hedis [15], Hello [22], Searchlight [13] and U-Connect [18] (we also implemented Quorum [24], but the result is only presented in Figure 9 since it is inapplicable when the users’ duty cycle are different), and run these algorithms in a cluster with nine servers, each equipped with an Intel Xeon 2.6 GHz CPU (central processing unit) with 24 hyper-threading cores, 64 GB memory and 1T SSD (solid-state disk). The basic settings in the simulations are: $d_{c}=50$ m, $P_{max}=100,000,p_{n}=1$ and $t_{0}=20$ ms. We choose three scenarios for comparison:

(1) Discovery in a star network. The central node $u_{c}$ has $|N_{c}|$ neighbors in the star network. A neighboring node selects the duty cycle randomly within [0.1,0.5], while $u_{c}$’s duty cycle ($\theta_{c}$) is set to different figures.
(2) Discovery among N=1000 nodes. The area is set as a rectangle of size 1000 × 1000 m${}^{2}$, and the node’s coordinates are generated randomly. Each node selects the duty cycle randomly within [0.1,0.5].
(3) Discovery between two neighbors. Spear enables the evaluation for existing protocols and generates the best schedule for fixed duty cycles.

We evaluated average discovery latency, lifetime, or percentage of discovery under different settings, and we describe the detailed parameters for each figure. The start time of any node is generated randomly within [0,1000] and the results are based on 1000 separate runs. The detailed parameters are described in Table 3.

### 7.1. Increasing Discovery Rate

In the network with multiple nodes, communication collisions could affect the discovery results. We evaluate the performance of the proposed collision reducing methods (PPR and DPR in Section 6), and compare them with the bare (not running in Spear) algorithms.

**Figure 4:**
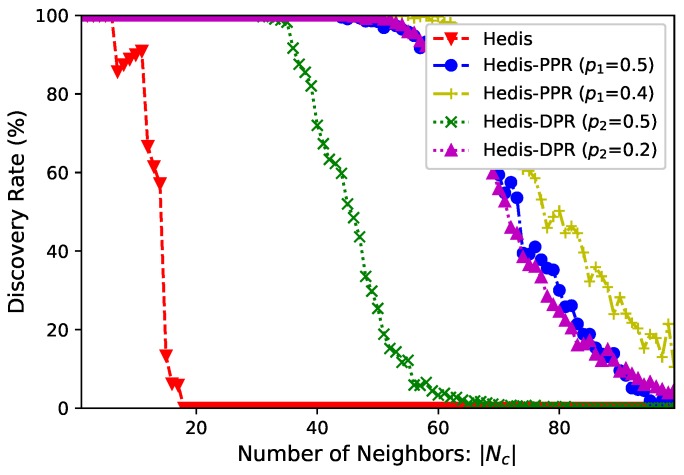
Spear increases discovery rate by incorporating PPR and DPR.

**Number of neighbors**. In a star network, the central node $u_{c}$ has $|N_{c}|$ neighbors and it selects duty cycle $\theta_{c}=0.3$. We select Hedis as the example and set $p_{1}=p_{2}=0.5$ for PPR and DPR. Figure 4 shows the discovery rate (*y*-axis) of $u_{c}$ within 100,000 time slots when $|N_{c}|$ (*x*-axis) increases from 1 to 100. From the figure, (bare) Hedis cannot discover all neighbors when $|N_{c}|\geq 7$, and it cannot find even one neighbor when $|N_{c}|\geq 18$. Modified by PPR and DPR, all neighbors can be discovered when $|N_{c}|\leq 43$ and $|N_{c}|\leq 33$, respectively. We also evaluate the performance of PPR and DPR at $p_{1}=0.4$ and $p_{2}=0.2$, and they outperform the methods when $p_{1}=p_{2}=0.5$.

**Figure 5:**
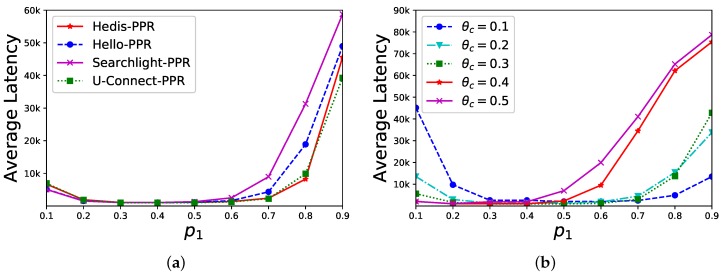
Sensitivity of $p_{1}$ in the PPR method. (**a**) $\theta_{c}$ = 0.3; (**b**) U-Connect-PPR.

**Sensitivity of $p_{1}$**. In a star network, the central node $u_{c}$ has $|N_{c}|=20$ neighbors (we set $|N_{c}|=20$ since the bare algorithms fail to find any neighbor) and we evaluate PPR’s performance under different values of $p_{1}$. As shown in Figure 5a, $\theta_{c}$ is set to 0.3 and the average discovery latency (*y*-axis) of different algorithms is changed by different values of $p_{1}$ (*x*-axis). When $p_{1}\in \left[0.3,0.4\right]$, the performance is better. In Figure 5b, we select U-Connect as the example and set $\theta_{c}$ as 0.1,0.2,0.3,0.4,0.5, respectively. The average discovery latency is changed by different values of $p_{1}$ (*x*-axis), and the performance is also better when $p_{1}$ approaches [0.3,0.4]. Overall, our discovery latency is stable when $p_{1}\leq 0.6$.

**Figure 6:**
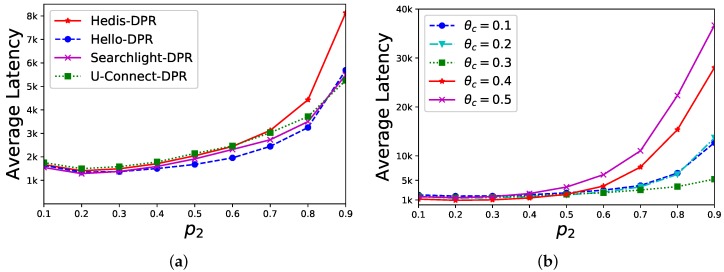
Sensitivity of $p_{2}$ in the DPR method. (**a**) $\theta_{c}$ = 0.3; (**b**) U-Connect-DPR.

**Sensitivity of $p_{2}$**. In a star network, the central node $u_{c}$ has $|N_{c}|=20$ neighbors and the DPR’s performance is evaluated under different values of $p_{2}$. As shown in Figure 6a, $\theta_{c}$ is set to 0.3 and the average discovery latency (*y*-axis) of different algorithms are changed by different values of $p_{2}$ (*x*-axis). When $p_{2}$ is close to 0.2, the performance is better. In Figure 6b, we select U-Connect for different $\theta_{c}$ (0.1,0.2,0.3,0.4,0.5, respectively). The average discovery latency (*y*-axis) is changed by different values of $p_{2}$ (*x*-axis), and the performance is better when $p_{2}\approx 0.2$. Overall, our discovery latency is stable when $p_{2}\leq 0.7$.

**1000 nodes**. In a randomly generated network with N=1000 nodes, we evaluate the performances of different algorithms. As shown in Figure 1, modified by PPR ($p_{1}=0.4$) and DPR ($p_{2}=0.2$), the discovery rates (*y*-axis) are much larger than those of the bare algorithms. Especially for Hello, the discovery rate is only 33.0%, while PPR and DPR could greatly increase the rate to 99.2%,95.5%, respectively.

### 7.2. Prolonging Lifetime

The two methods (PWR and PDR) that can extend a node’s lifetime are also implemented in Spear. We evaluate the performance in a star network where the central node $u_{c}$ has $|N_{c}|=20$ neighbors. In PWR, m=30 levels of remaining energy and corresponding duty cycles are generated in advance. In PDR, ${\hat{\theta }}_{0}$ is set to 0.3, $P_{min}=200$, and the node adjusts the duty cycle every $\hat{T}=100,000$ time slots.

**Figure 7:**
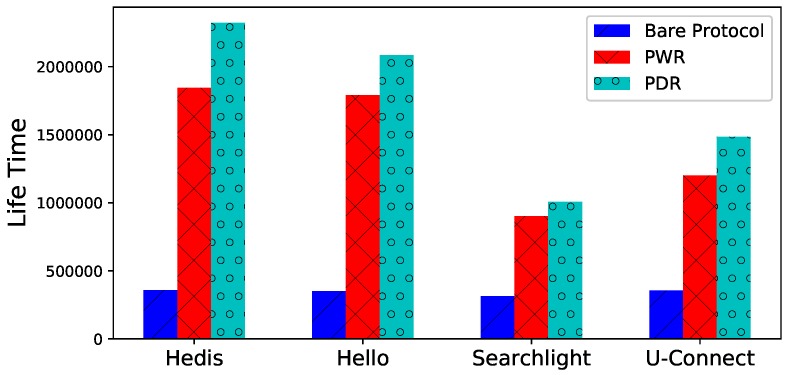
Spear prolongs the lifetime with PWR and PDR.

**Lifetime**. The lifetime of $u_{c}$ is illustrated in Figure 7 for different algorithms, both PWR and PDR prolong the lifetime significantly. For example, the lifetimes of PWD and PDR are 5.15 and 6.47 times longer than bare Hedis, respectively.

**Figure 8:**
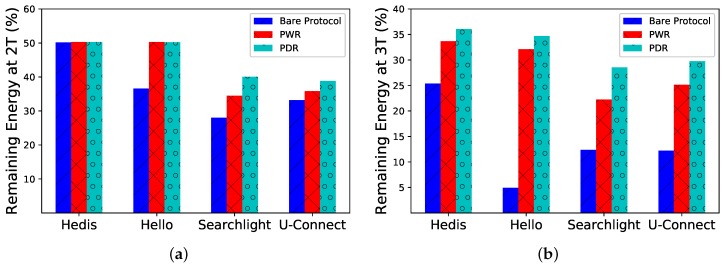
Spear saves more energy by incorporating PWR and PDR compared to bare algorithms. (**a**) After $2\hat{T}$; (**b**) After $3\hat{T}$.

**Percentage of Remaining Energy**. We show the percentage of remaining energy (*y*-axis) after $2\hat{T}$ and $3\hat{T}$ time slots in Figure 8. After $2\hat{T}$ time slots, PWR and PDR are just a little better than the bare Hedis protocol (see Figure 8a), while the difference becomes much larger after $3\hat{T}$ time slots (see Figure 8b). By incorporating PWR and PDR, Spear can save power and extend the lifetime significantly.

### 7.3. Improving Discovery for Two Neighbors

Spear enables the evaluation of neighbor discovery protocols supporting symmetric and asymmetric duty cycles, which facilitates the generation of the optimal schedule.

**Figure 9:**
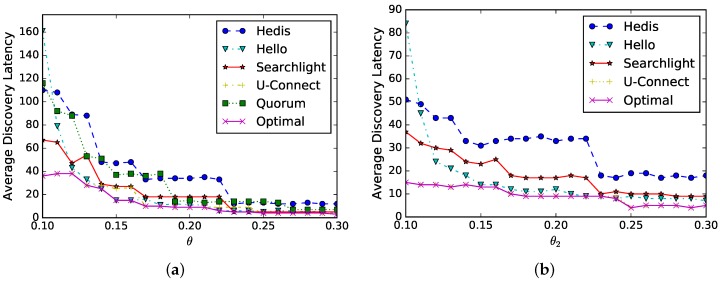
Spear enables the evaluation of the neighbor discovery algorithms for symmetric and asymmetric duty cycles and generates the optimal schedule automatically. (**a**) Symmetric duty cycle; (**b**) asymmetric duty cycle.

**Symmetric duty cycle**: suppose two neighbors select the same duty cycle θ that increases from 0.1 to 0.3, we evaluate the average discovery latency in Figure 9a. As shown in the figure, the discovery latency (*y*-axis) of all algorithms decreases as θ (*x*-axis) increases, and they have similar performances. This is because the discovery latency is proportional to $\frac{1}{\theta^{2}}$ and the trends of these curves match the analysis. Finally, the optimal schedule can be generated automatically; for example, U-Connect is selected when θ∈[0.1,0.13], while Hello is selected when θ∈[0.13,0.16].

**Asymmetric duty cycle**: suppose one node’s duty cycle is fixed as $\theta_{1}=0.2$ and the other node’s duty cycle $\theta_{2}$ increases from 0.1 to 0.3. Since Quorum is inapplicable for asymmetric duty cycles, we compare the other four algorithms. As shown in Figure 9b, the average discovery latency (*y*-axis) decreases as $\theta_{2}$ (*x*-axis) increases, and the decreasing trends are much more gentle than in Figure 9a. This is because the discovery latency is proportional to $\frac{1}{\theta_{2}}$ when $\theta_{1}$ is a constant.

### 7.4. Effectiveness of Spear Components

In Spear, the communication module evidently increases the discovery rate as compared to the bare protocols, as demonstrated by the evaluations in Section 7.1. The energy module significantly extends a node’s lifetime, as described in Section 7.2. Though we did not evaluate the application module separately, the evaluations targeting at discovery latency and discovery rate imply that Spear could adjust the related parameters (such as duty cycle, $p_{1}$ of PPR, and $p_{2}$ of DPR) to reduce the discovery latency. The algorithmic module computes an optimal schedule automatically as shown in Section 7.3.

## 8. Conclusions

We present Spear, the first practical framework for general neighbor discovery protocols in WSNs. Extensive evaluations have shown that Spear can greatly increase the discovery rate and extend the lifetime of sensor nodes. Spear has the potential to be applied to tackling a broad range of problems in WSNs.

In the future, we would like to explore the connection between some standard MAC (media access control) protocols for reducing collisions and neighbor discovery protocols, such as the Carrier Sense multiple Access/Collision Avoidance (CSMA/CA) protocol. We would also compare the performance of the proposed methods with the standards and bring these intuitive ideas to design efficient neighbor discovery protocols. Furthermore, we would evaluate the proposed framework with some well known IoT operation systems such as RiOT and Contiki [7], and conduct experiments on a real deployed WSN.

## Figures and Tables

**Table 1:** Notations for Neighbor Discovery.

Notation	Description
$u_{i}$	Sensor node $u_{i}$
$I_{i}$	Identifier of node $u_{i}$
$P_{max}$	The maximum energy of each sensor
$P_{i}\left(t\right)$	The remaining energy of $u_{i}$ at time *t*
$t_{0}$	The length of each time slot
$p_{n}$	The consumed energy to turn on the radio in each slot
$d_{c}$	Communication range of each sensor
$t_{i}^{s}$	Start time of node $u_{i}$
$S_{i}$	Neighbor discovery schedule of node $u_{i}$
$s_{i}\left(t\right)$	The schedule of node $u_{i}$ at time *t*
L(i,j)	Discovery latency between $u_{i},u_{j}$
$N_{i}$	The set of neighbors of node $u_{i}$
$L(i,N_{i})$	The latency for $u_{i}$ to discover all neighbors
$\theta_{i}\left(T_{1},T_{2}\right)$	Node $u_{i}$’s duty cycle between time $\left[T_{1},T_{2}\right]$
$t_{i}^{e}$	The time node $u_{i}$ runs out of energy
$Lf_{i}$	Lifetime of node $u_{i}$

**Table 2:** Algorithms comparison for two neighbors.

Algorithms	DC 1	DC 2	Latency	Asymmetric?
Quorum [24]	θ	θ	$\frac{4}{\theta^{2}}$	No
LL-Optimal [33]	θ	θ	$\frac{1}{\theta^{2}}$	No
Disco [16]	$\theta_{1}$	$\theta_{2}$	$\frac{4}{\theta_{1}\theta_{2}}$	Yes
U-Connect [18]	$\theta_{1}$	$\theta_{2}$	$\frac{9}{4\theta_{1}\theta_{2}}$	Yes
Searchlight [13]	$\theta_{1}$	$\theta_{2}$	$\frac{2}{\theta_{1}\theta_{2}}$	Yes
C-Torus [35]	$\theta_{1}$	$\theta_{2}$	$\frac{9}{4\theta_{1}\theta_{2}}$	Yes
BlindDate [26]	$\theta_{1}$	$\theta_{2}$	$\frac{9}{5\theta_{1}\theta_{2}}$	Yes
Hedis [15]	$\theta_{1}$	$\theta_{2}$	$\frac{4}{\theta_{1}\theta_{2}}$	Yes
Todis [15]	$\theta_{1}$	$\theta_{2}$	$\frac{9}{\theta_{1}\theta_{2}}$	Yes
Hello [22]	$\theta_{1}$	$\theta_{2}$	$\frac{\left(c_{1}+1\right)\left(c_{2}+1\right)}{c_{1}c_{2}\theta_{1}\theta_{2}}$	Yes

**Remarks:** (1) ‘DC’ is short for duty cycle; we use θ for symmetric duty cycle and $\theta_{1},\theta_{2}$ for asymmetric duty cycle; (2) Hello is a little different from other algorithms, where there are two parameters to choose; (3) some results of discovery latency are modified (or simplified) on the basis of symmetric analyses.

**Table 3:** Parameters of the evaluations.

Figures	Parameters			
All figures	$d_{c}=50$ m	$P_{max}=$ 100,000	$p_{n}=1$	$t_{0}=20$ ms
Figure 4	$|N_{c}|\in \left[1,100\right]$	$\theta_{c}=0.3$	$p_{1}=0.5$	$p_{2}=0.5$
Figure 5	$|N_{c}|=20$	$\theta_{c}=0.3$	$p_{1}\in \left[0.1,0.9\right]$	
Figure 6	$|N_{c}|=20$	$\theta_{c}=0.3$	$p_{2}\in \left[0.1,0.9\right]$	
Figure 7	$|N_{c}|=20$	${\hat{\theta }}_{0}=0.3$	$\hat{T}=$ 100,000	
Figure 8	$|N_{c}|=20$	${\hat{\theta }}_{0}=0.3$	$\hat{T}=$ 100,000	
Figure 9	N=2	$\theta_{1}\in \left[0.1,0.3\right]$	$\theta_{2}\in \left[0.1,0.3\right]$	
